# Antimalarial and antitumour activities of the steroidal quinone-methide celastrol and its combinations with artemiside, artemisone and methylene blue

**DOI:** 10.3389/fphar.2022.988748

**Published:** 2022-09-02

**Authors:** Jerome P. L. Ng, Yu Han, Li Jun Yang, Lyn-Marie Birkholtz, Dina Coertzen, Ho Ning Wong, Richard K. Haynes, Paolo Coghi, Vincent Kam Wai Wong

**Affiliations:** ^1^ Neher’s Biophysics Laboratory for Innovative Drug Discovery, State Key Laboratory of Quality Research in Chinese Medicine, Macau University of Science and Technology, Macau, China; ^2^ Department of Biochemistry, Genetics and Microbiology, University of Pretoria Institute Malaria for Sustainable Malaria Control, University of Pretoria, Hatfield, South Africa; ^3^ Centre of Excellence for Pharmaceutical Sciences, School of Health Sciences, North-West University, Potchefstroom, South Africa; ^4^ School of Pharmacy, Macau University of Science and Technology, Macau, China

**Keywords:** celastrol, redox drug, artemisinin, artemisone, synergism, malaria, cancer

## Abstract

Artemisinin, isolated from the traditional Chinese medicinal plant qīng hāo 青蒿 (*Artemisia annua*) and its derivatives are used for treatment of malaria. With treatment failures now being recorded for the derivatives and companion drugs used in artemisinin combination therapies new drug combinations are urgently required. The amino-artemisinins artemiside and artemisone display optimal efficacies *in vitro* against asexual and sexual blood stages of the malaria parasite *Plasmodium falciparum* and are active against tumour cell lines. In continuing the evolution of combinations of the amino-artemisinins with new drugs, we examine the triterpenoid quinone methide celastrol isolated from the traditional Chinese medicinal plant léi gōng téng 雷公藤 (*Tripterygium wilfordii*). This compound is redox active, and has attracted considerable attention because of potent biological activities against manifold targets. We report that celastrol displays good IC_50_ activities ranging from 0.50–0.82 µM against drug-sensitive and resistant asexual blood stage *Pf*, and 1.16 and 0.28 µM respectively against immature and late stage *Pf* NF54 gametocytes. The combinations of celastrol with each of artemisone and methylene blue against asexual blood stage *Pf* are additive. Given that celastrol displays promising antitumour properties, we examined its activities alone and in combinations with amino-artemisinins against human liver HepG2 and other cell lines. IC_50_ values of the amino-artemisinins and celastrol against HepG2 cancer cells ranged from 0.55–0.94 µM. Whereas the amino-artemisinins displayed notable selectivities (SI > 171) with respect to normal human hepatocytes, in contrast, celastrol displayed no selectivity (SI < 1). The combinations of celastrol with artemiside or artemisone against HepG2 cells are synergistic. Given the promise of celastrol, judiciously designed formulations or structural modifications are recommended for mitigating its toxicity.

## 1 Introduction

**FIGURE 1:**
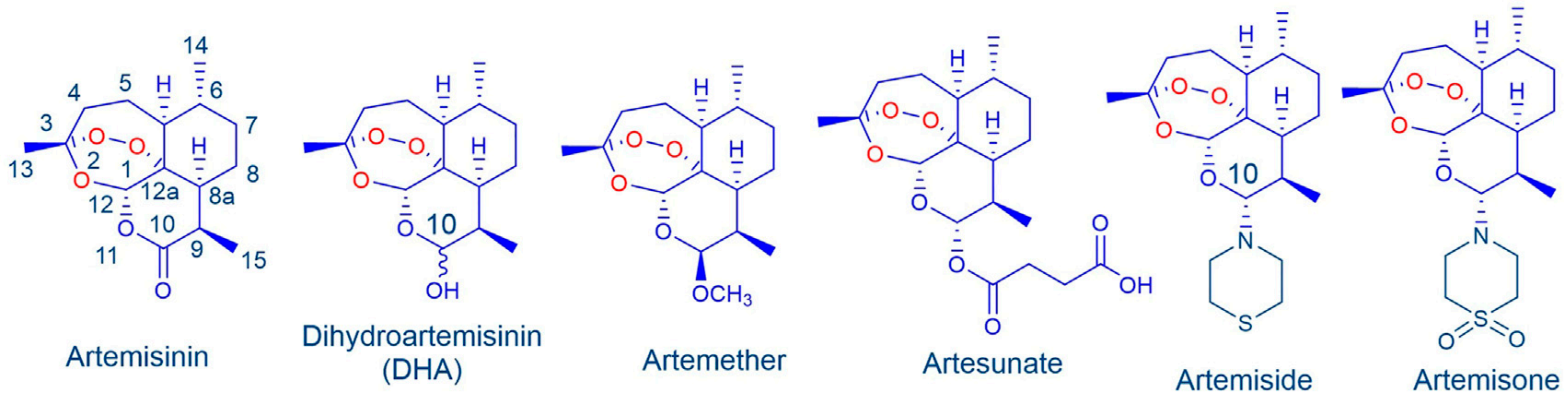
Artemisinin and clinical derivatives DHA, artemether and artesunate. The amino-artemisinins artemiside and artemisone possess a nitrogen atom attached to C-10 that enhances biological activities.

Treatment of malaria with artemisinin combination therapies (ACTs) comprising artemisinin or one of its clinical derivatives (Figure 1) (Cui and Su, 2009; Li et al., 2017) with a longer half-life antimalarial drug (Eastman and Fidock, 2009; Wells et al., 2009; World Health Organisation, 2021) is compromised by enhanced tolerance of the malaria parasite, principally *Plasmodium falciparum* (*Pf*), to the artemisinin, and resistance to the partner drug (Phyo and von Seidlein, 2017; Woodrow and White, 2017; Ouji et al., 2018; Mairet-Khedim et al., 2021). The increased tolerance to artemisinins is due to induction of dormancy (quiescence) in ring stage parasites in response to drug pressure. Whilst various explanations are put forward for quiescence (Mbengue et al., 2015; Siriwardana et al., 2016), the most likely cause is an enhanced stress response (Bridgford et al., 2018; Rocamora et al., 2018; Wellems et al., 2020). New drug combinations based on rational consideration of mechanism of action of the components are urgently required. Here we focus on amino-artemisinins (Haynes et al., 2004) that have enhanced efficacies against all blood stages of the malaria parasite. The best-known is artemisone (Figure 1) (Haynes et al., 2006; Vivas et al., 2007). Its potent biological activities are ascribed to the properties of the amino group at C-10 (Wu et al., 2016), improved pharmacokinetics (Nagelschmitz et al., 2008), and generation of active metabolites with relatively long half-lives (Schmeer et al., 2005; Nagelschmitz et al., 2008; Gibhard et al., 2021; Watson et al., 2021).

**FIGURE 2:**
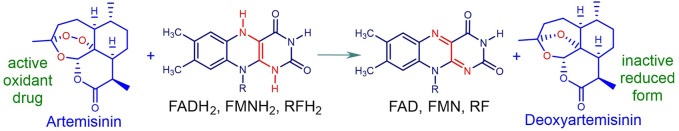
Oxidation of reduced flavins by artemisinin: FAD flavin adenine dinucleotide, FMN flavin mononucleotide, RF riboflavin. Formal two-electron transfer results in irreversible reduction of the artemisinin to deoxyartemisinin. Thereby, scavenging of electrons from the reduced flavin within the flavin disulfide reductase e.g., GR, TrxR, lipoamide dehydrogenase and others results in blockade of GSH supply. Abrupt build-up of reactive oxygen species (ROS) upon addition of the artemisinin that may be associated with induction of downstream signalling pathways results in enhanced oxidative stress (*cf.* Figure 3 below).

In order to select the combination partner, one needs to briefly consider mechanism of action (MoA) of artemisinins. Whilst the thesis involving artemisinin activation by heme is the most strongly supported (Giannangelo et al., 2019; Quadros et al., 2022), there are difficulties in reconciling this with experimental observations, known aspects of free radical chemistry, and in particular structure-activity relationships of artemisinin derivatives and analogues (Haynes et al., 2004; Haynes et al., 2013). We have shown that these compounds rapidly oxidize reduced flavin cofactors of the disulfide reductases glutathione reductase (GR), thioredoxin reductase (TrXR) and others important for maintaining redox homeostasis in the malaria parasite. The artemisinin is thereby irreversibly reduced and enhancement of oxidative stress ensues (Figure 2) (Haynes et al., 2010; Haynes et al., 2011; Haynes et al., 2012).

**FIGURE 3:**
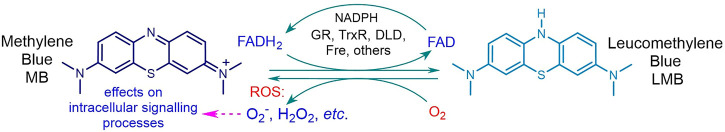
Reduction of MB by reduced cofactor flavin adenine dinucleotide FADH_2_ to LMB and reoxidation by O_2_ to regenerate MB. Scavenging of electrons by MB from FADH_2_ within the flavoenzyme disulfide reductase *e.g.* glutathione reductase GR, thioredoxin reductase TrxR, dihydrolipoamide dehydrogenase DLD, results in abrogation of supply of GSH or other biogenic thiol, and sustained build-up of ROS, with futile consumption of NADPH.

In order to enhance the stress response, a drug capable of redox cycling is best used in combination with the artemisinin. One such is methylene blue (MB) (Figure 3), active against asexual blood stage malaria parasites and displaying synergism with artemisinins (Akoachere et al., 2005; Buchholz et al., 2008). MB also has gametocytocidal activity (Adjalley et al., 2011) that is synergized by artemiside and artemisone (Coertzen et al., 2018; Wong et al., 2019). Notably, MB rapidly oxidizes the same reduced flavin cofactors that are oxidized by the artemisinins and is thereby reduced to leucomethylene blue (LMB). The latter is reoxidized by oxygen to MB and the ensuing redox cycling results in build-up of ROS. Thus, NADPH that regenerates FADH_2_ from FAD undergoes futile consumption (Figure 3) (Haynes et al., 2012). The redox cycling of MB will promote the action of artemisinins (Figure 3).

Artemisinins also show antitumour activities (Efferth, 2017; Konstat-Korzenny et al., 2018; Augustin et al., 2020; Kiani et al., 2020; Mancuso et al., 2021). Artemisone elicits activities superior to artemisinin *in vitro* (Gravett et al., 2011; Das, 2015). The activities (IC_50_) against tumour cell lines range from 0.26–95.7 µM (Gravett et al., 2011; Hooft van Huijsduijnen et al., 2013; Dwivedi et al., 2015; Wu et al., 2018; Wong et al., 2020). As in the case of malaria, MoA likely involves interruption of function of flavin cofactors of disulfide reductases, leading to generation of intracellular ROS that through downstream signalling events overwhelms redox homeostasis in the cancer cell (Efferth, 2017; Wong et al., 2020). Other ROS-independent pathways may also be involved (Qin et al., 2015; Greenshields et al., 2019). Combinations of artemisinins with known and experimental cancer drugs have been examined (Efferth, 2017; Kumar et al., 2019; Koltai, 2021). Artemisone shows additivity in combinations with oxaliplatin and gemcitabine *in vitro* (Gravett et al., 2011). Artemisone is also active against A375 melanoma cells (IC_50_ 95.7 µM), wherein synergism with the redox-active copper (II) complex of the anticancer drug elesclomol is observed. Further, generation of ROS is demonstrated, and use of flow cytometry in combination with the FITC Annexin V assay indicates the artemisinin induces apoptotic cell death, that is greatly enhanced in the presence of the companion drug (Wong et al., 2020). Thus, the case for continuing the examination of combinations of the amino-artemisinins with redox-active drugs to enhance cancer chemotherapy is clear.

**FIGURE 4:**
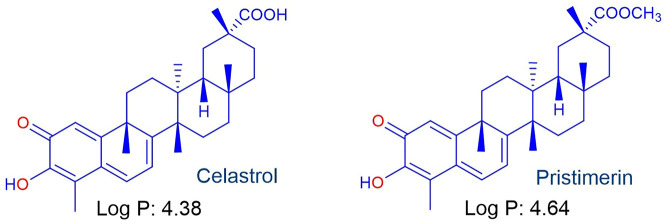
The lipophilic pentacyclic triterpene celastrol isolated from *Tripterygium wilfordii* used in Traditional Chinese Medicine and the naturally-occurring methyl ester pristimerin.

We now consider the lipophilic triterpenoid quinone-methide celastrol (Figure 4), also known as tripterine, isolated from the traditional Chinese medicinal plant léi gōng téng 雷公藤 or Thunder of God vine (*Tripterygium wilfordii*) as the redox component (Chen et al., 2018). Celastrol exhibits potent antitumour and other biological activities that involve *inter alia* redox activity associated with generation of intracellular ROS and modulation of downstream signalling pathways (Moreira et al., 2019; Peng et al., 2019; Chen et al., 2020; Lu et al., 2021; Youns et al., 2021). Celastrol also inhibits the flavoenzyme siderophore A that catalyzes the hydroxylation of L-ornithine in *Apergillis fumigatus*. Whilst *in silico* experiments suggested reversible binding at the active site of siderophore A (Martín Del Campo et al., 2016), the effect of celastrol on the redox cycling of the flavin cofactor (*cf.* Effect of MB, Figure 3) was not considered. However, that quinone-methides are redox active and rapidly oxidize reduced flavin cofactors in flavoenzymes is illustrated by the behaviour of the quinone oxidoreductases NQO1 and NQO2 upon treatment with *o-* and *p*-quinone methides derived from *o*- and *p-*cresol respectively (Kucera et al., 2013). Thus, we anticipate as for MB (Figure 3) (Coertzen et al., 2018; Wong et al., 2019), celastrol should synergize the action of artemisinins against different targets. However, although celastrol is a biologically potent compound, it displays untoward toxicity associated with numerous off-target effects. Thus, considerable effort has been directed towards development of controlled-release formulations of celastrol (Huang., et al., 2020; Shi et al., 2020; Guo et al., 2021; Wagh et al., 2021) or preparation of less toxic derivatives largely associated with conversion of the carboxylic acid to amide derivatives (Klaić et al., 2012; Bassanini et al., 2021; Coghi et al., 2021).

Antimalarial activities of celastrol and its naturally-occurring methyl ester pristimerin (Figure 4) have been reported (Figueiredo et al., 1998; Li et al., 2019); for celastrol, IC_50_ activities against the chloroquine sensitive *Pf* NF54 strain and multidrug resistant *Pf* K1 strain are 564 and 401 nM respectively, with similar values for pristimerin (IC_50_ NF54 583 nM, K1 409 nM). Thus far, the antimalarial MoA has not been elucidated. However, it is clear that the redox-active quinone methide moiety of celastrol bestows overall biological activity (Kucera et al., 2013; Moreira et al., 2019; Peng et al., 2019; Chen et al., 2020; Lu et al., 2021; Youns et al., 2021). In this sense, this aspect correlates with the antimalarial activities of MB and redox-active naphthoquinones such as menadione (Lanfranchi et al., 2012; Klotz et al., 2014) that are active against asexual and sexual blood stage parasites (Gupta et al., 2002; Tanaka et al., 2015; Ehrhardt et al., 2016; Sidorov et al., 2016; Ahenkorah et al., 2020).

Here, we present the results of an evaluation of the antimalarial activity *in vitro* of celastrol alone and in combination with each of artemisone and MB using standardized screens (Coertzen et al., 2018; Wong et al., 2019). Likewise, antitumour activities of each of artemiside, artemisone and celastrol individually and in combination against selected cancer cell lines according to reported methods are described (Wong et al., 2020; Ng et al., 2022).

## 2 Materials and methods

### 2.1 Materials

Reference compounds and the artemisinins used for screening were ≥95% pure (Coertzen et al., 2018; Wong et al., 2019; Gibhard et al., 2021). Celastrol purchased from the Chengdu SanHerb BioScience company (Chengdu, China), was ≥95% pure, and was used without further purification.

### 2.2 Antimalarial efficacies

#### 2.2.1 Antimalarial efficacies

*In vitro cultivation of asexual and gametocyte P. falciparum parasites*: *P. falciparum* asexual blood stage parasites were cultivated in human erythrocytes in RPMI-1640 media supplemented with AlbuMax II under sterile conditions and in a hypoxic environment (90% N_2_, 5% CO_2_, and 5% O_2_) at 37°C as described previously (Coertzen et al., 2018; Wong et al., 2019). Gametocytes were produced from asexual blood stage parasites in a stage-specific manner through induction of cellular stress as described previously (Reader et al., 2015; Coertzen et al., 2018; Wong et al., 2019; Reader et al., 2022).

#### 2.2.2 Asexual blood stage parasites

DHA, artesunate, artemether, chloroquine (CQ), and methylene blue (MB) were used as reference drugs. All assay conditions are as previously described (Coertzen et al., 2018; Wong et al., 2019). Compound working solutions were prepared from a 10 mM stock solution in 100% (v/v) dimethyl sulfoxide (DMSO; Sigma-Aldrich) in AlbuMAX II supplemented RPMI 1640 medium with a final DMSO concentration of 0.1% (v/v), shown to be nontoxic to be nontoxic to intraerythrocytic asexual blood stage parasites (Coertzen et al., 2018). Dose-responses were determined using a 2-fold serial drug dilution on *in vitro* 95% ring-stage intraerythrocytic *Pf* parasites (1% parasitemia, 1% hematocrit) at 37°C under 90% N_2_, 5% CO_2_, and 5% O_2_ atmospheric conditions, detecting SYBR green I fluorescence as a marker for parasite proliferation following a 96 h drug treatment (Smilkstein et al., 2004; Verlinden et al., 2011). Activity against the *Pf* drug-sensitive NF54 strain and the multi drug-resistant K1 (resistant to CQ, quinine, pyrimethamine, and cycloguanil), and W2 (resistant to CQ, quinine, pyrimethamine, and cycloguanil) strains was evaluated. Untreated and 1 µM CQ-treated parasites were included as positive and negative controls. MB and artemisone were included as internal reference standards. Data analysis was performed using GraphPad Prism (version 6) software, intra-assay variability was monitored with Z-factors, and acceptable inter-assay reproducibility was determined from the percent coefficient of variation (CV) (Reader et al., 2015). Data are from technical triplicates, performed for three biological replicates. Results are expressed as the compound concentration at which 50% parasite viability/proliferation is affected (IC_50_).

#### 2.2.3 Immature and late-stage gametocytes

Gametocytocidal activity was determined using the transgenic NF54-*Pfs16*-GFP-Luc reporter lines (Adjalley et al., 2011; Reader et al., 2015; Reader et al., 2022) to derive dose responses and determine IC_50_ after 48 h continuous drug pressure against immature gametocytes (2-fold serial drug dilutions on ≥95% stage II-III gametocytes) or after both 48 and 72 h continuous drug pressure against more mature late-stage IV - V gametocytes (≥90% stages IV and V, 10-fold serial drug dilutions) (2%–3% gametocytemia, 2% hematocrit) at 37°C under hypoxic conditions. Untreated and 5 μM MB-treated immature and late-stage gametocytes were included as positive and negative controls. MB and artemisone were included as internal reference standards. In all cases, an interference assay to eliminate false positives from possible compound interference with the luciferase readout was run in parallel. Unless otherwise indicated, data are from technical triplicates, performed for three biological replicates. Complete dose-response curves are given in the (Supplementary Figures S2A,B).

#### 2.2.4 Drug combination assays against asexual blood stage parasites

The *in vitro* interactions of celastrol with artemisone and MB was determined using a fixed-ratio isobole analysis on *Pf* NF54 asexual parasites (SYBR Green I-based fluorescence). Briefly, the drugs were applied alone at their respective IC_50_ value and in fixed-drug percentage combination ratios of IC_50_ values of 100:0, 80:20, 60:40, 40:60, 20:80, and 0:100, two-fold serially diluted and grown for 96 h at 37°C under the 90% N_2_, 5% O_2_, and 5% CO_2_ gas mixture in 96-well plates to obtain the IC_50_ dose response curves for each drug alone and in the fixed-drug ratio (Ohrt et al., 2002; Fivelman et al., 2004). The fractional inhibitory concentration (FIC) for each drug in the combination was calculated as follows:

FIC = IC_50_ of drug A in combination with drug B/IC_50_ of drug A

The paired FIC values for the drugs in each combination were linearly plotted to provide the isobologram. The ΣFIC of FIC of drug A in combination with FIC of drug B was determined by calculating the mean FIC value, to obtain the representative FIC value for the drug combination. Data obtained were analysed in Excel, and sigmoidal dose-response curves and isobolograms were plotted using GraphPad 6.0. Experiments were performed in triplicate, and repeated 3 times.

### 2.3 Cytotoxicity

Proliferative and non-proliferative mammalian cell lines: human liver and lung cancer cell lines HepG2 and A549, and immortalized normal liver LO2 and lung BEAS-2B cells were purchased from ATCC (Manassas, VA, United States). Cells were cultured in RPMI-1640 medium supplemented with 10% fetal bovine serum and antibiotics penicillin (50 U/mL) and streptomycin (50 μg/ml; Invitrogen, United Kingdom). All cells were incubated at 37°C in a 5% humidified CO_2_ incubator.

#### 2.3.1 Assays

All test compounds were dissolved in DMSO at a final concentration of 50 mM and stored at −20°C before use. Cytotoxicity was assessed with A549, HepG2, BEAS-2B and LO2 cells. The MTT assay with 3-(4,5-dimethylthiazole-2yl)-2,5-diphenyltetrazolium bromide (5 mg/ml) was performed as previously described (Ng et al., 2022). Briefly, 4 × 10^3^ cells per well were seeded in 96-well plates before drug treatment. After overnight cell culture, the cells were exposed to different concentrations of selected compounds (0.19–100 μM) for 72 h. Cells without drug treatment were used as control. Next, 10 μl of the MTT solution was added to each well and incubated at 37°C for another 4 h. Solubilization buffer (100 μl) was then added (10 mM HCl in a solution of 10% of SDS) and incubated overnight. The absorbance A at 570 nm was measured on the next day. The percentage of cell viability was calculated using the following formula: Cell viability (%) = A_treated_/A_control_ × 100. Dose response curves for all assays are given in the Supplementary Figures S3–S6.

#### 2.3.2 Drug combination assays

For the drug combination inhibitory assays, six drug preparations, of which four comprised combinations of either artemiside or artemisone with celastrol in a fixed ratio of 80:20, 60:40, 40:60, and 20:80 were prepared and screened against HepG2 liver hepatocellular carcinoma and LO2 human normal hepatocytes respectively. Two of these six preparations employed each of artemiside, artemisone or celastrol alone at a concentration approximately 5–7 times higher than the IC_50_ of the individual drug as presented in Table 4. For artemiside, 500 µM was taken as 5-fold IC_50_ and for celastrol 20 µM was taken as 7-fold IC_50_. Thus, six combinations for artemiside (µM) and celastrol (µM) prepared were 500:0, 400:4, 300:8, 200:12, 100:16, and 0:20, respectively.

**FIGURE 5:**
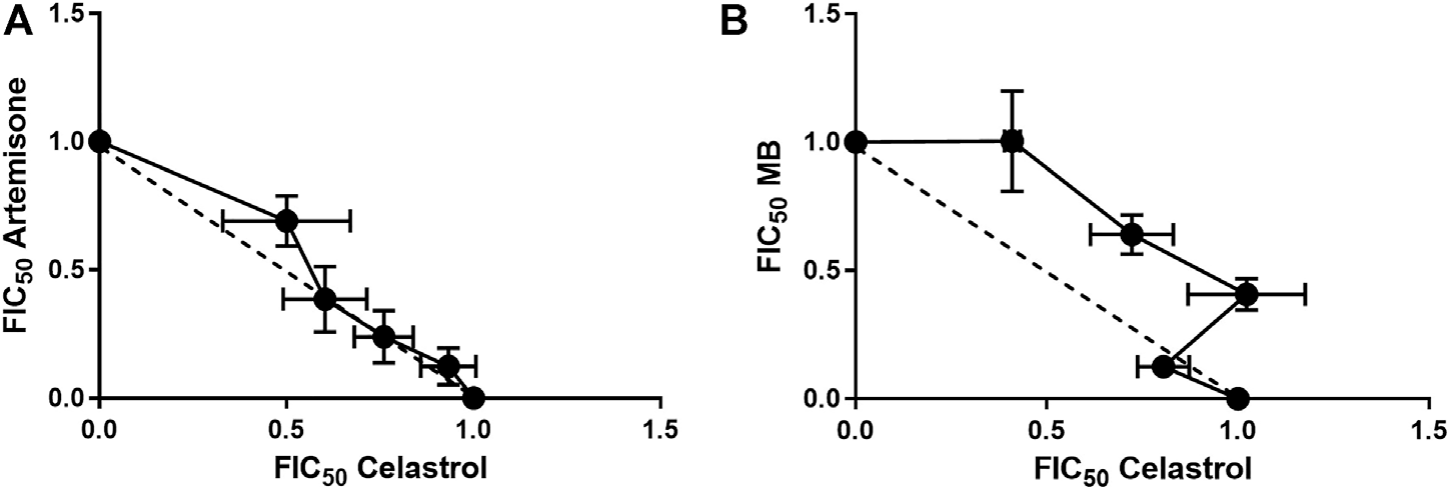
Isoboles for artemisone and MB in combination with celastrol. Isobole analysis was performed for **(A)**: artemisone in combination with celastrol and **(B)** MB in combination with celastrol against asexual blood stage NF54 parasites using the SYBR Green I based assay. Results are representative of four independent biological replicates (n = 4), each performed in technical triplicates, mean ± SEM.

As above for the malaria combination assays, a mean ΣFIC<1.0 represents a synergistic interaction, >1.3 represents an antagonistic interaction and ΣFIC = 1 represents an indifferent or additive interaction. Additivity, synergism and antagonism were also be established from the linear plots constructed from the FIC values of each of the drugs. A concave hyperbolic plot indicates synergism, a convex hyperbolic plot indicates an antagonistic interaction, and a straight line indicates an additive interaction. Isobole analysis of the combinations of each of artemiside and artemisone in combination with celastrol was performed against HepG2 and LO2 cells respectively. In Figure 5 is presented the isoboles for each drug combination.

## 3 Results and discussion

### 3.1 Antimalarial efficacy

#### 3.1.1 Asexual blood stage parasites

Activity was determined using the SYBR Green I based fluorescence assay on asexual blood stages of the NF54 (drug sensitive), K1 and W2 (drug resistant) strains of *P. falciparum*. In Table 1 are presented the activities and the resistance index (RI) for each drug resistant strain. Dose response curves are given in the Supplementary Figures S1A–C. Also included in Table 1 for direct comparison of activities against celastrol are data for the internal reference standards MB and artemisone recorded at the same time as for celastrol. The activities correlate with the data previously reported for these compounds including the other artemisinin derivatives of Table 1 (Coertzen et al., 2018; Wong et al., 2019).

**TABLE 1:** Activities (nM) *in vitro* against asexual blood stage *P. falciparum*.

^a^Compound	IC_50_ nM^b^				
	NF54	K1	RI^c^	W2	RI^d^
Methylene Blue^f^	5.9 ± 0.8^e^/5.0 ± 0.8^f^	6.45 ± 0.30^f^	1.29^f^	5.13 ± 0.31^f^	1.03^f^
DHA^f^	2.51 ± 0.19	1.51 ± 0.33	0.6	1.74 ± 0.22	0.7
Artemether^f^	1.86 ± 0.17	9 ± 2	4.8	7 ± 1	3.8
Artesunate^f^	3.00 ± 0.29	4 ± 1	1.3	2.4 ± 0.4	0.8
Celastrol	820 ± 190	700 ± 100	0.85	500 ± 100	0.61
Artemiside^f^	1.11 ± 0.17	1.6 ± 0.4	1.47	1.75 ± 0.27	1.58
Artemisone^f^	2.32 ± 0.76^e^/1.2 ± 0.4^f^	1.01 ± 0.19^f^	0.85^f^	1.6 ± 0.4^f^	1.36^f^

a Structures of artemisinins in Figure 1, of celastrol in Figure 4; *P. falciparum* NF54 CQ, sensitive; K1: CQ, pyrimethamine, mefloquine, cycloguanil resistant; W2: CQ, quinine, pyrimethamine, cycloguanil resistant

b Results for proliferative (SYBR, Green I) assays from three biological replicates, each performed as technical triplicates, mean ± SEM

c Resistance index (RI) = IC_50_ K1/IC_50_ NF54

d IC_50_ W2/IC_50_ NF54

e data for internal reference standards, this study

f data from Coertzen et al., 2018; Wong et al., 2019.

Interestingly, celastrol displays antimalarial activities somewhat similar to those previously recorded (IC_50_ *Pf* NF54 564 nM, *Pf* K1 401 nM) using the tritiated hypoxanthine assay (Li et al., 2019), confirming that activities of celastrol are orders of magnitude inferior to those of the artemisinins. Whilst the activities in terms of IC_50_ data are better than those of the naphthoquinone menadione recorded using the tritiated hypoxanthine assay (IC_50_ 9.6–12 µM) (Lanfranchi et al., 2012), there are more recent examples of redox active naphthoquinones displaying superior activities against asexual blood stages of drug-sensitive and -resistant *Pf* (Verlinden et al., 2011; Ehrhardt et al., 2016; Sidorov et al., 2016; Ahenkorah et al., 2020; Ng et al., 2022).

#### 3.1.2 Stage specific gametocyte activity

Stage-specific activities of celastrol against immature (stage II-III) and late stage (stage IV-V) gametocytes were determined with the luc reporter line as described previously (Coertzen et al., 2018; Wong et al., 2019). Data are presented in Table 2. Also included in Table 2 for direct comparison of activities with celastrol are gametocytocidal data for MB, artemisone and the artemisinin derivatives recorded previously (Coertzen et al., 2018; Wong et al., 2019). Dose response curves are presented in the Supplementary Figures S2A,B. Although celastrol displayed relatively mediocre activity against immature stage gametocytes, it showed good activity against late stage IV-V gametocytes. This approximately 4-fold greater activity against late stage gametocytes is noteworthy, and in this respect, is more active than or equipotent with naphthoquinones against this stage for which comparable data have been obtained, such as for plasmodione (IC_50_ 1,107 nM) (Ehrhardt et al., 2016) and a series of imidazolo-naphthoquinones (IC_50_ 164–1,088 nM) (Ahenkorah et al., 2020).

**TABLE 2:** Activities (nM) *in vitro* against immature stage II-III and late stage IV-V *P. falciparum* NF54 gametocytes.

^a^Compound	^b^Immature stage II-III (luc 48 h) IC_50_ nM	^c^Late stage IV-V (luc 72 h) IC_50_ nM	Fold change preference ratio EG to LG	Fold change preference ratio LG to EG
Methylene Blue^d^	95.0 ± 11.3	143.0 ± 16.7	1.5	0.7
DHA^d^	43.0 ± 3.9	33.66 ± 1.98	0.78	1.3
Artemether^d^	37.7 ± 2.0	136.2 ± 85.9	3.6	0.28
Artesunate^d^	62.8 ± 3.0	259.4 ± 80	4.1	0.24
Celastrol	1,160 ± 66.5	282.4 ± 96.2	0.2	4.1
Artemiside^d^	16.4 ± 1.0	1.5 ± 0.5	0.09	10.9
Artemisone^d^	1.94 ± 0.11	42.4 ± 3.3	21.9	0.05

a Structures of artemisinins in Figure 1, of celastrol in Figure 4; IC_50_ values against

b immature stage II-III, gametoctyes (>90%)

c late stage IV-V, gametocytes (>90%) determined using the luciferase based assay against the Luc reporter cell line; results are representative of three biological replicates (n = 3), each performed in technical triplicates, mean ± SEM; data are from 48 h for immature gametocytes and 72 h for late stage IV-V, gametocytes drug incubation period

d data from Coertzen et al., 2018, Wong et al., 2019.

#### 3.1.3 Antimalarial drug combination studies

As previously recorded for each of artemiside and artemisone in combination with MB (Coertzen et al., 2018), drug-drug interactions were monitored through evaluation of fractional inhibitory concentration (FIC) as determined by isobolograms. A mean ΣFIC<1.0 represents a synergistic interaction, >1.3 an antagonistic interaction and ΣFIC = 1 an indifferent or additive interaction. Isobologram analysis of the combinations of each of artemisone and MB in combination with celastrol was performed against the asexual stages of *Pf* NF54 using the SYBR Green I based assay. In Table 3 are shown the calculated FIC values for the independent combinations at each ratio as well as the ∑FIC values for each combination. In Figure 5 are presented the independent isoboles for each drug combination.

**TABLE 3:** FIC values for artemisone and MB in combination with celastrol against asexual blood stage *Pf* NF54.^a^

Drug ratio	FIC values					
	Artemisone	Celastrol	ΣFIC	MB	Celastrol	ΣFIC
80:20	0.69	0.50	1.19	0.79	0.41	1.06
60:40	0.39	0.60	0.99	0.66	0.72	1.39
40:60	0.24	0.76	1.00	0.67	1.02	1.73
20:80	0.12	0.93	1.06	0.70	0.81	0.81
Avg ΣFIC			**1.06**			**1.25**

a Calculated FIC, and ΣFIC (highlighted in bold) values following isobole analysis of each of artemisone and methylene blue MB, in combination with celastrol. Results are representative of four independent biological repeats (n = 4), each performed as technical triplicates, ± SEM.

The combination of each of artemisone and MB with celastrol showed additive interactions as is apparent from the isobolograms (Figure 5) as well as the ΣFIC values for these combinations, with an average ΣFIC of 1.06 (95% confidence interval (CI) 94.91–95.09) for artemisone with celastrol and an average ΣFIC of 1.25 (95% CI 94.61–95.39) for MB with celastrol, similar to that previously observed for artemisone and MB (ΣFIC of 1.14) on asexual blood stage parasites (Coertzen et al., 2018).

### 3.2 Antitumor activities

Cytotoxicity was assessed for each of artemisinin, DHA and artemether, and the amino-artemisinins artemiside and artemisone, and celastrol against A549 human lung carcinoma, BEAS-2B non-tumorigenic human bronchial epithelium, HepG2 liver hepatocellular carcinoma and LO2 human normal hepatocytes using the MTT assay (Ng et al., 2022). Data are presented in Table 4, and dose response curves are given in the Supplementary Figures S3–S6.

**TABLE 4:** Cytotoxicities of artemisinins and celastrol (µM) against tumour and non-proliferating cell lines *in vitro*.

^a^Compound	IC_50_ µM^b^					
	A549	BEAS-2B	^c^SI	HepG2	LO2	^c^SI
Artemisinin	>100	>100	—	>100	>100	—
DHA	62.6 ± 3.4	21.2 ± 1.4	0.3	>100	30.7 ± 2.2	<0.3
Artemether	>100	>100	—	1.02 ± 0.2	>100	>98
Artemiside	>100	32.1 ± 2.7	<0.3	0.55 ± 0.02	>100	>183
Artemisone	>100	73.6 ± 1.4	<0.7	0.58 ± 0.2	>100	>171
Celastrol	2.83 ± 0.12	0.45 ± 0.1	0.16	0.94 ± 0.1	0.78 ± 0.2	0.83

a Structures of artemisinins in Figure 1, of celastrol in Figure 4.

b A549 human lung carcinoma; BEAS-2B, non-tumorigenic human bronchial epithelium; HepG2 liver hepatocellular carcinoma; LO2 human normal hepatocyte; results are reported as inhibitory concentrations IC_50_ from three independent biological replicates, each performed as technical replicates ±standard deviation (SD).

c SI, selectivity index IC_50_ normal cell line/IC_50_ tumour cell line.

The standout features are the relative toxicity of DHA towards the normal cell lines (SI < 1), and the selective activities of artemiside and artemisone against liver hepatocellular carcinoma cell lines (SI > 183 and >171 respectively), compared with celastrol which displays no selectivity with respect to normal hepatocytes (SI < 1, Table 4) (Klaić et al., 2012).

**FIGURE 6:**
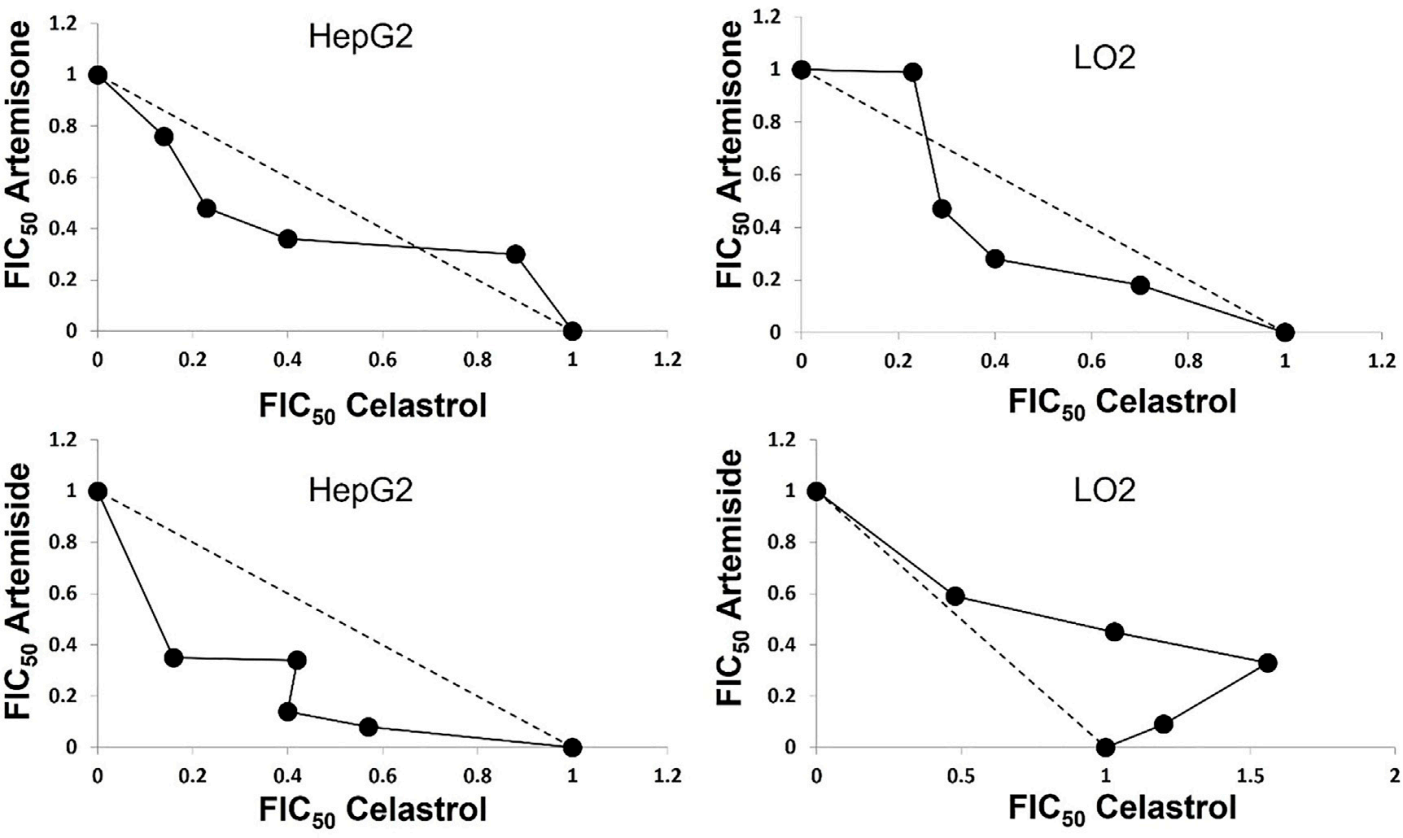
Isobole analyses of combinations of each of artemiside and artemisone with celastrol against HepG2 liver hepatocellular carcinoma and LO2 human normal hepatocyte cell lines; results are representative of four independent biological repeats (n = 4), each performed as technical triplicates, ± SEM.

Next, combinations of each of artemiside and artemisone with celastrol were examined, according to the method used to establish additivity/synergism described above. Interestingly, the mean of ΣFIC<1.0 for assays against HepG2 carcinoma cell lines indicates each of artemiside and artemisone display synergism with celastrol. Against the normal hepatocyte LO2 cell line, whilst the interaction of artemisone with celastrol was synergistic, that of artemiside was additive. Synergism and additivity were also evident from the linear plots determined from the FIC values of each of the drugs (Table 5 and Figure 6). Thus, synergism is confirmed for combinations of the amino-artemisinins with celastrol against the HepG2 carcinoma cell line, and for artemisone-celastrol against human normal hepatocyte LO2.

**TABLE 5:** Cytotoxicity FIC values for artemiside and artemisone in combination with celastrol.^a^

Cell line	Drug ratio	FIC values					
		Artemiside	Celastrol	ΣFIC	Artemisone	Celastrol	ΣFIC
HepG2	80:20	0.57	0.08	0.65	0.76	0.14	0.90
	60:40	0.4	0.14	0.54	0.48	0.23	0.71
	40:60	0.42	0.34	0.76	0.36	0.4	0.76
	20:80	0.16	0.35	0.51	0.3	0.88	1.18
	Avg ΣFIC			**0.61**			**0.89**
LO2	80:20	0.59	0.48	1.07	0.99	0.23	1.22
	60:40	0.45	1.03	1.48	0.47	0.29	0.76
	40:60	0.33	1.56	1.89	0.28	0.4	0.68
	20:80	0.09	1.2	1.29	0.18	0.7	0.88
	Avg ΣFIC			**1.43**			**0.88**

a Calculated FIC, and ΣFIC (highlighted in grey) values following isobole analysis of each of artemiside and artemisone in combination with celastrol. Results are representative of four independent biological repeats (n = 4), each performed as technical triplicates, ± SEM; HepG2 liver hepatocellular carcinoma, LO2 human normal hepatocytes.

## 4 Conclusion

The activities of the oxidant amino-artemisinins artemiside and artemisone as well as the redox active drug celastrol were determined against *P. falciparum* blood-stage asexual drug sensitive NF54 and multidrug resistant K1 and W2 parasite strains. It is demonstrated that the combinations of each of artemisone and of MB with celastrol are additive, with a final ΣFIC of 1.06 for artemisone with celastrol and a final ΣFIC of 1.25 for MB with celastrol. Therefore, for the asexual blood stage parasites, the results for the artemisone-celastrol combination are similar to the artemisone-MB combinations recorded earlier, wherein an ΣFIC of 1.14 is observed (Coertzen et al., 2018). With respect to the oxidant mode of action of the artemisinin, this thus strongly supports the precept of common redox mechanistic pathways for MB and celastrol. That is, it is likely that celastrol exerts oxidative stress through attrition of reduced flavin cofactors associated with redox-active flavoenzymes such as glutathione reductase, thioredoxin reductase and others responsible for maintaining redox homeostasis in the malaria parasite. For the first time, the effect of celastrol against blood stage gametocytes was evaluated. In comparison with the gametocytocidal activities of known naphthoquinones, celastrol showed good activity against late stage gametocytes. The results provide substantial impetus for examining the antimalarial mechanism of action of celastrol. Thus, proposed future work will aim to more precisely define the redox activity of celastrol including a delineation of its actual effects on reduced flavin cofactors in relation to the behaviour of MB (cf. Figure 3) (Haynes et al., 2012) and of naphthoquinones, as discussed in the Introduction.

For the antitumour activities, the key features that emerge here are the selectivities of artemiside and artemisone (SI > 170) toward the HepG2 cancer cell line with respect to LO2 normal cell line. Selectivity towards hepatocellular carcinoma elicited by artemisinins both *in vitro* and *in vivo* have been recorded previously (Hou et al., 2008; Nandi et al., 2021) but here, an advantage is conferred by the relative lack of toxicity of artemisone especially with respect to the relatively neurotoxic DHA and its prodrugs artesunate and artemether (Schmeer et al., 2005; Haynes et al., 2006; Vivas et al., 2007; Nagelschmitz et al., 2008; Wu et al., 2016; Watson et al., 2021). Artemiside that is active against the HepG2 carcinoma cell line as noted here, and against malaria and other apicomplexan parasites is metabolized to artemisone and other active metabolites *in vivo* that in essence greatly extends the half-life of active drug and enhances overall bioavailability (Gibhard et al., 2021). Thus, the potential of artemiside to act as an antitumour agent requires further evaluation. In addition, we show here that drug interactions between each of artemiside and artemisone with celastrol are synergistic, thus supporting the strategy of combining the oxidant amino-artemisinin with the redox active celastrol. In this sense, the parallel in activities of the artemisone-celastrol combination with the artemisone - elesclomol-Cu as noted in the Introduction is apparent, and may involve a similar mechanistic pathway.

Overall, even though celastrol is so biologically active, its non-selectivity with respect to cytotoxicity towards normal cell lines is an issue, as has been noted on many occasions previously. Clearly, evaluation of selective formulation methods (Huang, T, et al., 2020; Shi et al., 2020; Guo et al., 2021; Wagh et al., 2021) or of relatively non-toxic derivatives (Klaić et al., 2012; Bassanini et al., 2021; Coghi et al., 2021) must continue in order to develop celastrol as a potent and successful drug.

## Data Availability

The original contributions presented in the study are included in the article/Supplementary Material, further inquiries can be directed to the corresponding authors.
