# Supplementary material for: Antimalarial and antitumour activities of the steroidal quinone-methide celastrol and its combinations with artemiside, artemisone and methylene blue
Source: Front Pharmacol. 2022 Sep 2;13:988748. doi: 10.3389/fphar.2022.988748 (PMC9479156; doi:10.3389/fphar.2022.988748)
Supplement: Supplementary file 1 [file DataSheet1.docx]

Supplementary Material

**
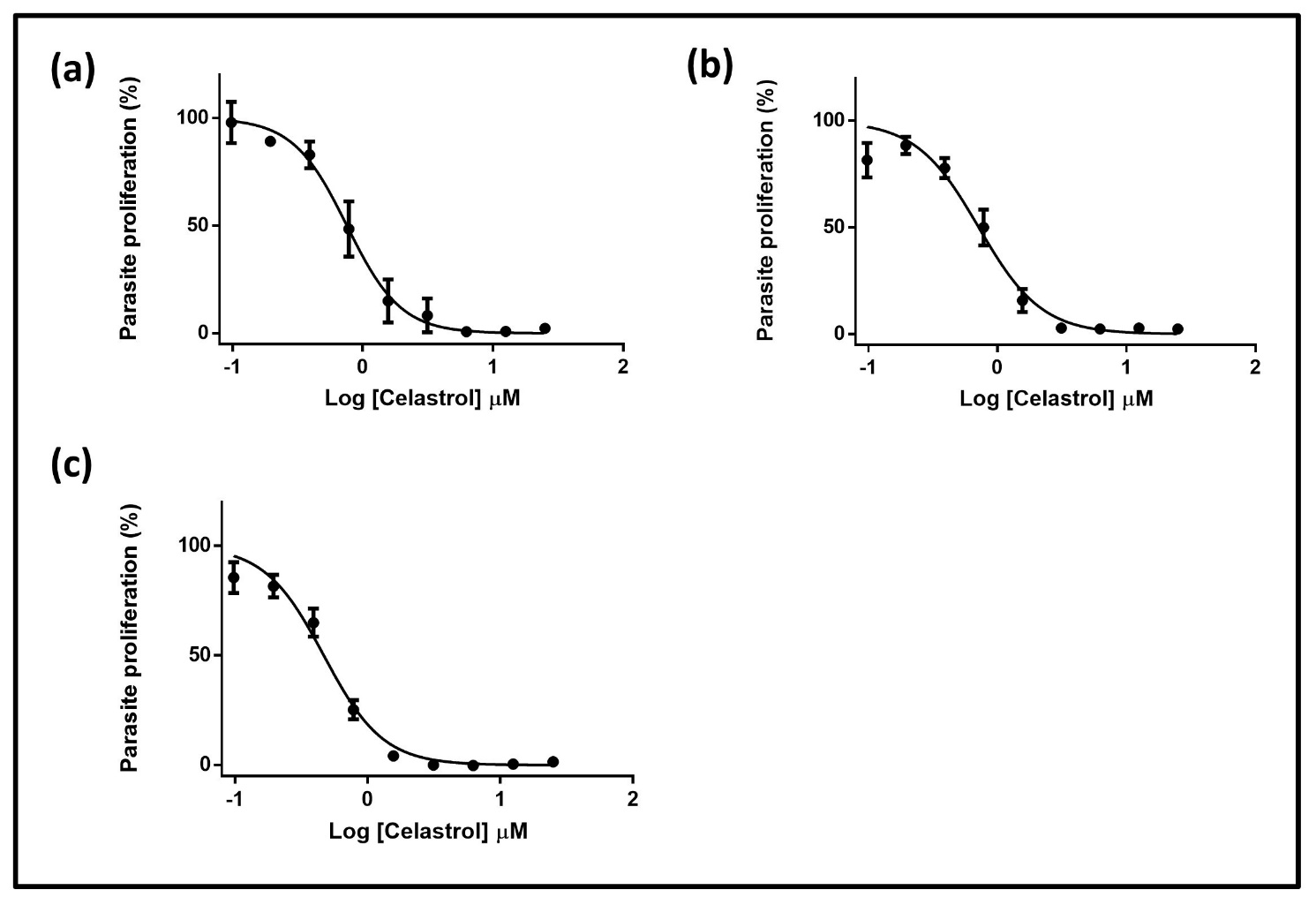
**

**Supplementary Figure S1.** Dose response curves for celastrol against against the asexual blood stage *Plasmodium falciparum* (*Pf*). **(a)** Dose response curve of celastrol against asexual blood stage *Pf* NF54 strain. Curves represents three independent biological repeats (n=3), error bars indicate SEM. **(b)** Dose response curve of celastrol against the asexual blood stage *Pf* K1 strain. Curves represents three independent biological repeats (n=3), error bars indicate SEM. **(c)** Dose response curve of celastrol against the asexual blood stage *Pf* W2 strain. Curves represents three independent biological repeats (n=3), error bars indicate SEM.

**
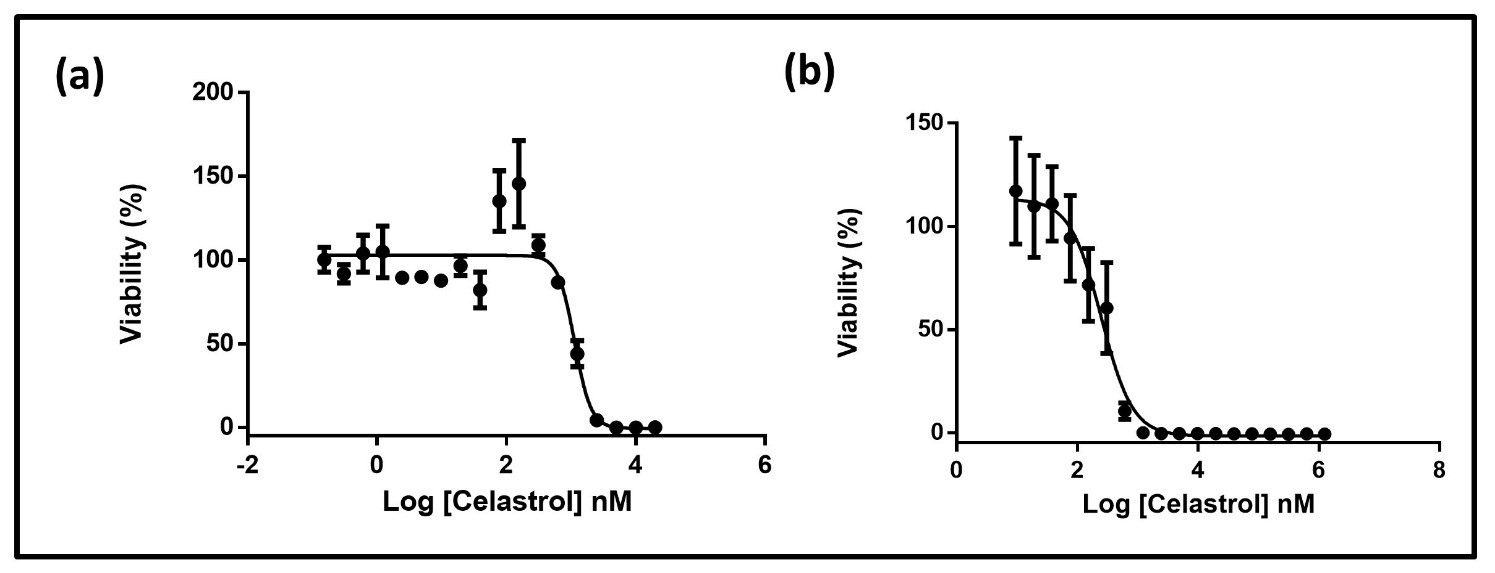
**

**Supplementary Figure S2.** Dose response curves for celastrol against early stage (EG) and late stage gametocytes of P.falciparum parasites. **(a)** Dose response curves of celastrol against EG determined with the luciferase assay. The curve represents three independent biological replicates performed in technical triplicates (n=3), error bars indicate SEM. **(b)** Dose response curve of celastrol against LG determined with the luciferase assay. The curve represents three independent biological replicates performed in technical triplicates (n=3), error bars indicate SEM.

**Cytotoxicity studies**

For compound **1** and **4** cytotoxicity data are consistent with those reported in literature from our group in (Yaremenko et al., 2020).

For compound **7** are consistent with those reported in literature from our group in (Coghi et al., 2021).


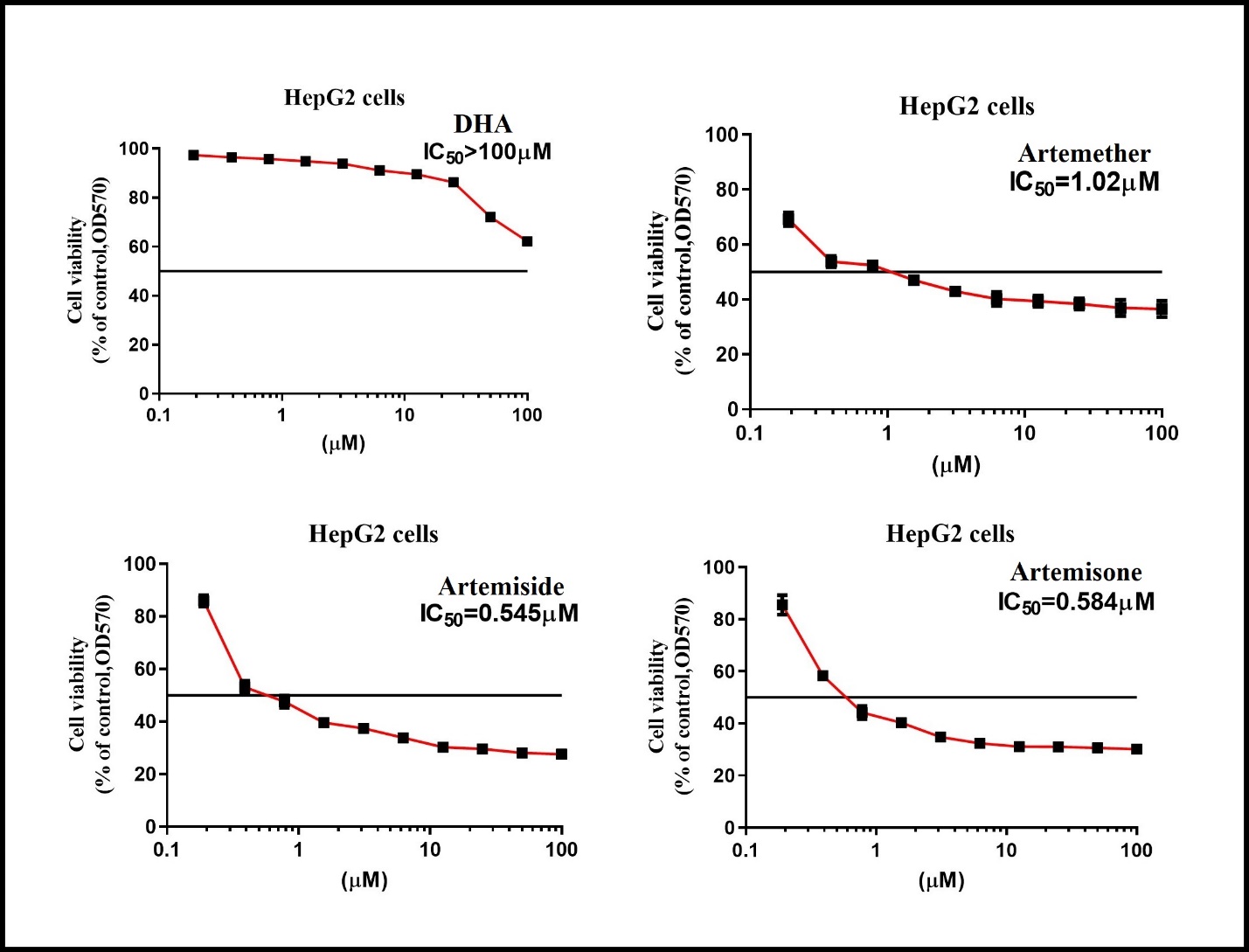


**Supplementary Figure S3.** Dose response curves of artemisinin derivatives, amino-artemisinins and celastrol against HepG2 cell line.


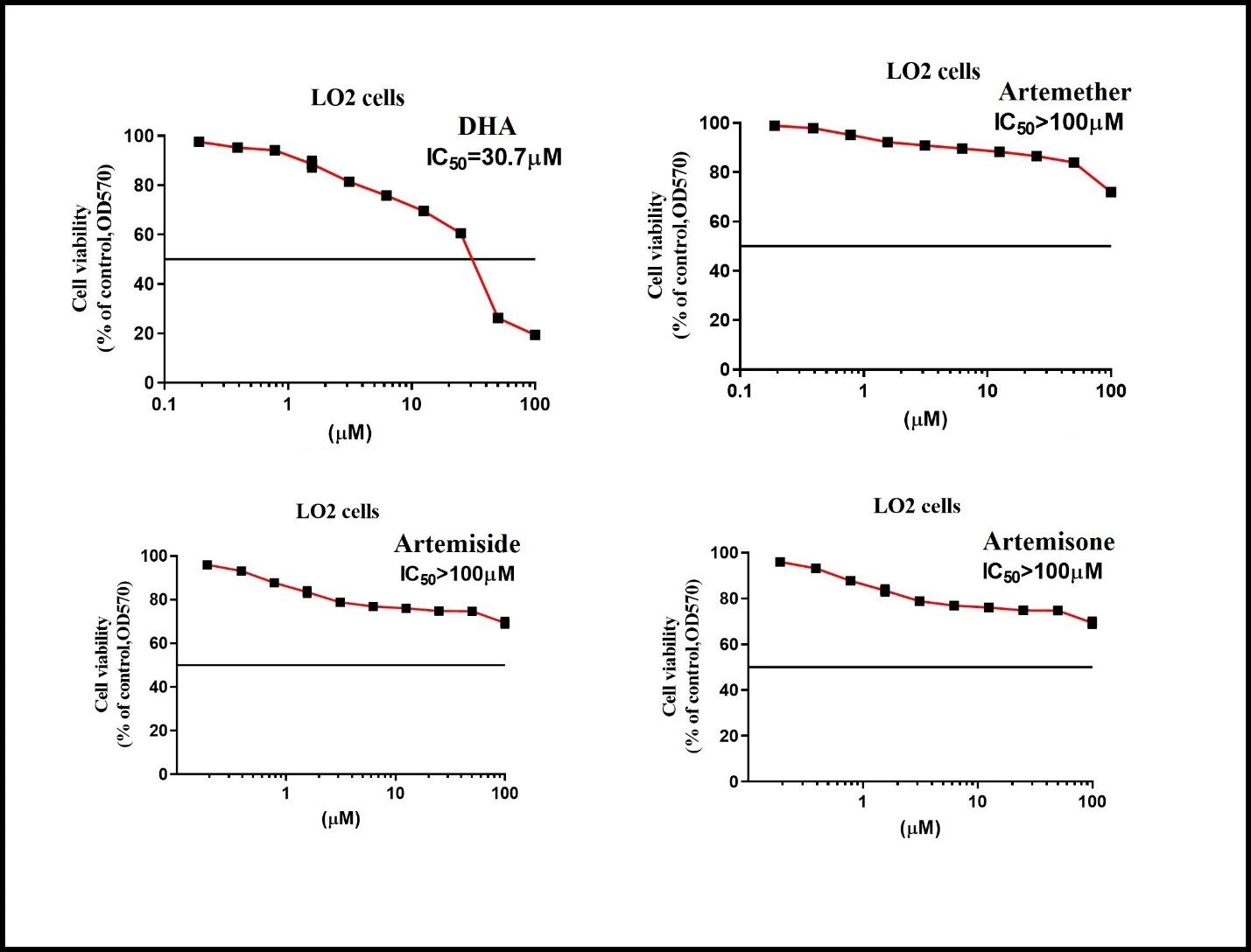


**Supplementary Figure S4.** Dose response curves of artemisinin derivatives, amino-artemisinins and celastrol against LO2 cell line.


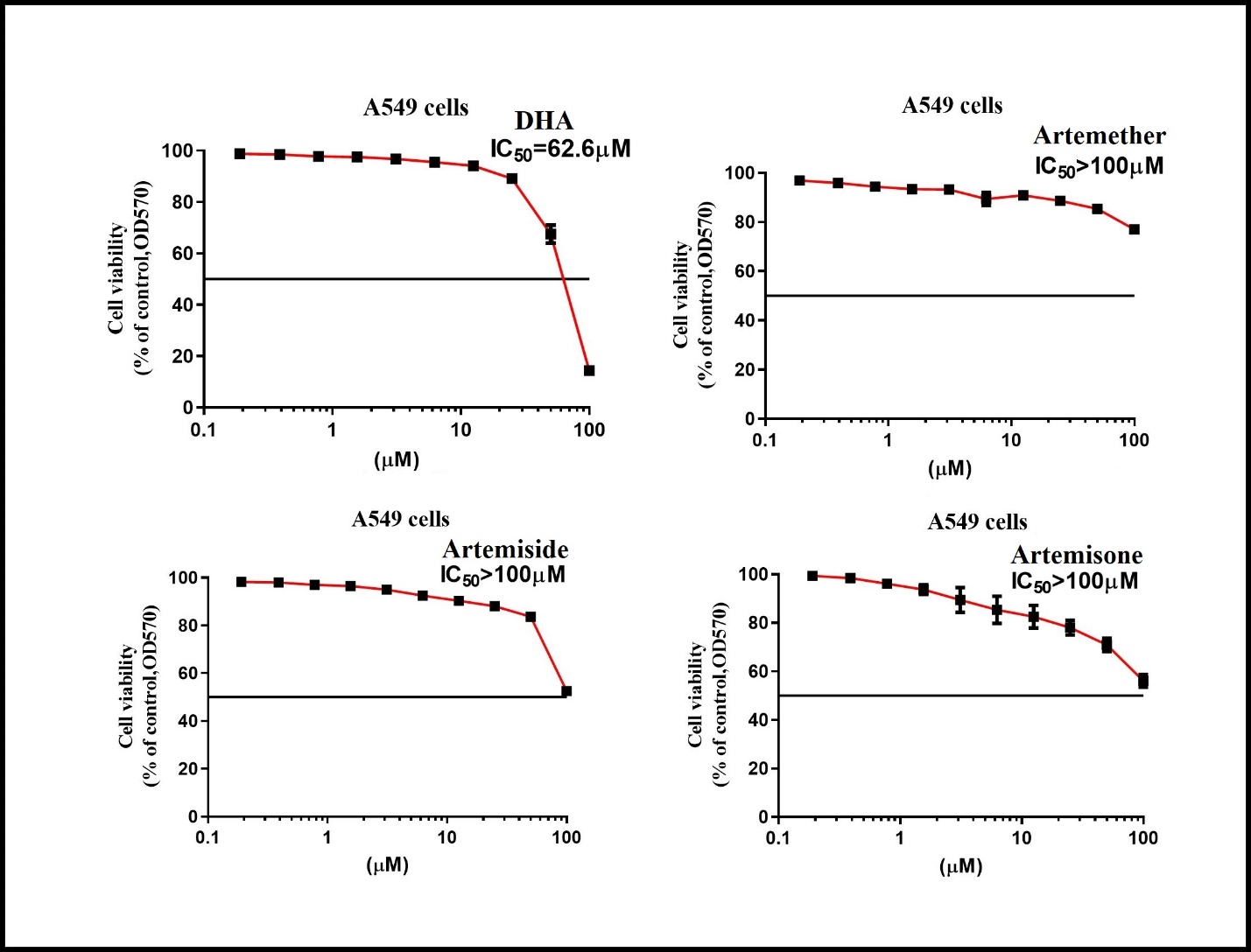


**Supplementary Figure S5.** Dose response curves of artemisinin derivatives, amino-artemisinins and celastrol against A549 cell line.


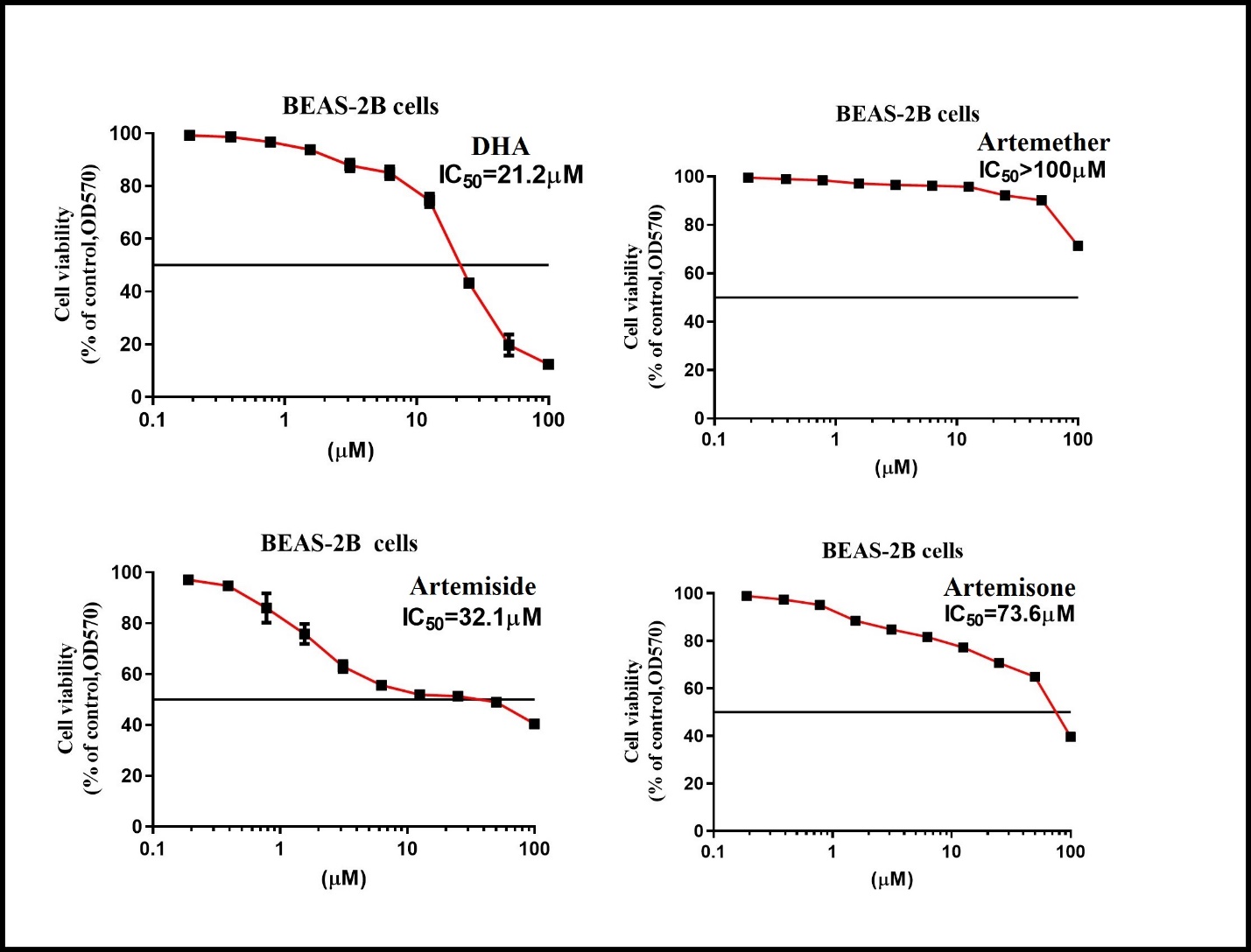


**Supplementary Figure S6.** Dose response curves of artemisinin derivatives, amino-artemisinins and celastrol against BEAS-2B cell line.

**References**

Yaremenko I. A., Coghi P., Prommana P., Qiu C., Radulov P. S., Qu Y., et al. (2020). Synthetic Peroxides Promote Apoptosis of Cancer Cells by Inhibiting P-Glycoprotein ABCB5. *ChemMedChem.* 15, 1118-1127. doi: 10.1002/cmdc.202000042

Coghi, P., Ng, J. P. L., Kadioglu, O., Law, B. Y. K., Qiu, A. C., Saeed, M. E. M., et al. (2021). Synthesis, computational docking and biological evaluation of celastrol derivatives as dual inhibitors of SERCA and P-glycoprotein in cancer therapy. *Eur. J. Med. Chem.* 224, 113676. doi: 10.1016/j.ejmech.2021.113676
